# Whole-Depth Change in Bovine Zona Pellucida Biomechanics after Fertilization: How Relevant in Hindering Polyspermy?

**DOI:** 10.1371/journal.pone.0045696

**Published:** 2012-09-26

**Authors:** Massimiliano Papi, Roberto Brunelli, Giuseppe Familiari, Maria Cristina Frassanito, Luciano Lamberti, Giuseppe Maulucci, Maurizio Monaci, Carmine Pappalettere, Tiziana Parasassi, Michela Relucenti, Lakamy Sylla, Fulvio Ursini, Marco De Spirito

**Affiliations:** 1 Istituto di Fisica, Università Cattolica del Sacro Cuore, Roma, Italy; 2 Dipartimento di Scienze Ginecologico-Ostetriche e Scienze Urologiche, Università di Roma Sapienza, Roma, Italy; 3 Dipartimento di Scienze Anatomiche, Istologiche, Medico-Legali e dell’Apparato locomotore, Università di Roma Sapienza, Roma, Italy; 4 Dipartimento di Ingegneria Meccanica e Gestionale, Politecnico di Bari, Bari, Italy; 5 Dipartimento di Patologia, Diagnostica e Clinica Veterinaria, Università di Perugia, Perugia, Italy; 6 Istituto di Farmacologia Traslazionale, CNR, Roma, Italy; 7 Dipartimento di Chimica Biologica, Università di Padova, Padova, Italy; 8 Fondazione di Ricerca e Cura Giovanni Paolo II, Fisica Sanitaria, Campobasso, Italy; VU University Medical Center, The Netherlands

## Abstract

Polyspermy is a common problem in bovine in vitro fertilization (IVF) and has a still unclear etiology. In this specie, after IVF, despite the lack of a biochemical post-fertilization hardening, the stiffness of the outer ZP layer is significantly increased. Therefore, polyspermy might be related to an incomplete or insufficient stiffening of the ZP. We obtained, by using atomic force spectroscopy in physiological conditions, a complete characterization of the biomechanical changes of the inner and outer ZP layers occurring during oocyte maturation/fertilization and correlated them to the ultrastructural changes observed by transmission electron microscopy using ruthenium red and saponin technique. In both the inner and outer ZP layers, stiffness decreased at maturation while, conversely, increased after fertilization. Contextually, at the nanoscale, during maturation both ZP layers displayed a fine filaments network whose length increased while thickness decreased. After fertilization, filaments partially recovered the immature features, appearing again shorter and thicker. Overall, the observed biomechanical modifications were substantiated by ultrastructural findings in the ZP filament mesh. In fertilized ZP, the calculated force necessary to displace ZP filaments resulted quite similar to that previously reported as generated by bovine sperm flagellum. Therefore, in bovine IVF biomechanical modifications of ZP appear ineffective in hindering sperm transit, highlighting the relevance of additional mechanisms operating in vivo.

## Introduction

The zona pellucida (ZP) is a porous network of sulphated glycoprotein filaments surrounding mammalian eggs [1]–[3], whose penetration by spermatozoa plays a crucial role in fertilization, with malfunctioning leading either to infertility or polyspermy. An increased resistance of the zona pellucida (ZP) to proteolytic digestion, the so called ZP hardening, induced soon after fertilization by the exocytosis of cortical granules has been interpreted as one of the mechanisms that concur to the block of polyspermy. However, this event, albeit common in mammals [4] does not occur in all species. Really, previous studies revealed that in vitro matured pig and cow oocytes do not undergo ZP hardening after IVF but rather experience a pre-fertilization hardening during their transit along the oviductal ampulla: this latter reaction hampers spermatozoa interaction with the oocyte due to the action of an oviduct-specific glycoprotein–heparin protein complex and therefore contributes to the prevention of polyspermy [5]. Lack of pre-fertilization ZP hardening [6] or, alternatively, a defective or incomplete granules release [7] during IVF protocols could explain the extremely high incidence of polyspermy in ungulates.

Of note, in all these previous reports, the above mentioned pre- and post-fertilization hardening – despite the term recalling the concept of an increased stiffness – were not correlated to a change in the biophysical properties of the ZP. By using atomic force spectroscopy (AFS), we recently demonstrated that, despite the lack of a biochemical post-fertilization hardening, the stiffness of the outer ZP layer is significantly increased after bovine oocyte IVF [8]. With reference to this evidence, we speculated that a stiffening of the ZP that was either incomplete, with regard to the ZP depth, or quantitatively insufficient, might concur to explain the high incidence of polyspermy during IVF. These two possibilities were investigated in the present study, as we examined whether changes in ZP biomechanics during in vitro maturation and fertilization: 1) occur throughout the ZP depth; 2) correlate with ultrastructural modifications of the protein filaments mesh; 3) can effectively cope with the recently reported force exerted by sperm flagellum. The results seriously question the putative importance of ZP biomechanics in preventing IVF-related polyspermy.

**Figure 1:**
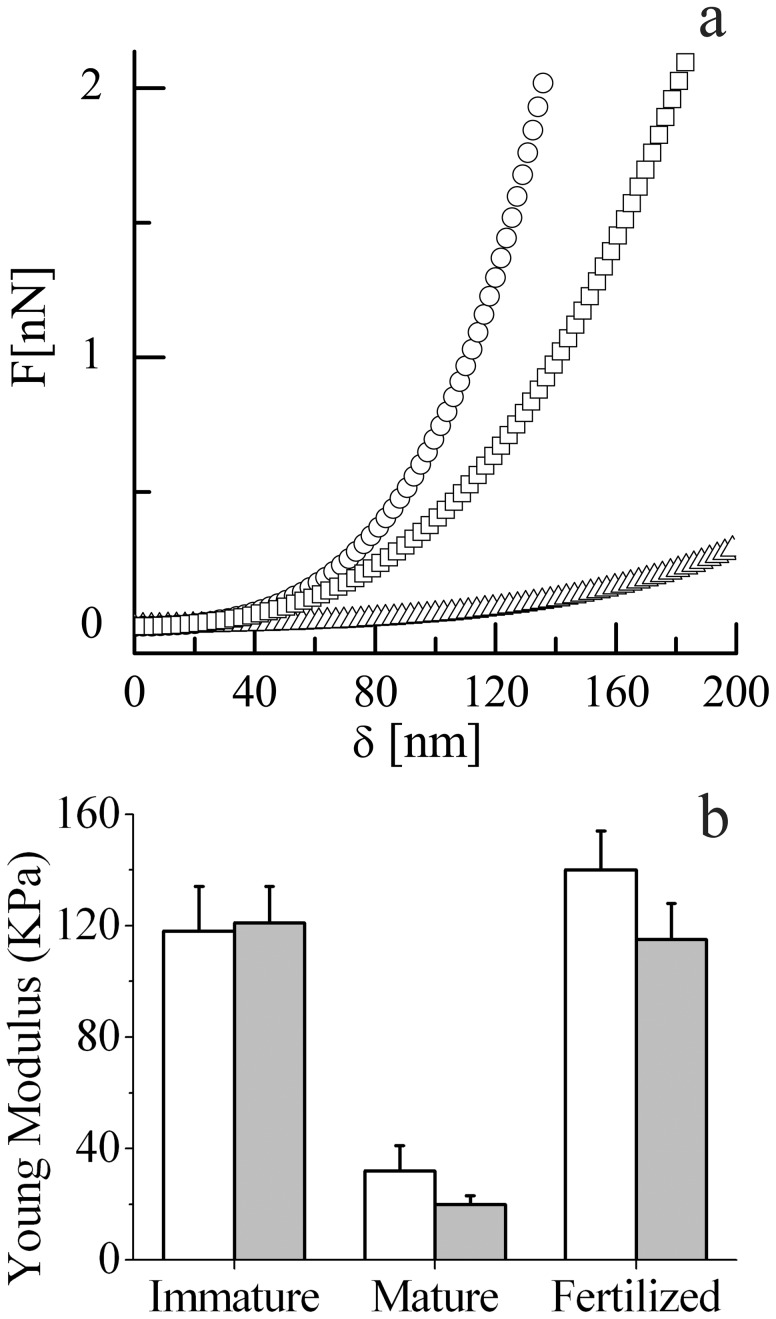
Biomechanical characterization of ZP. **a**: Representative force-indentation curves. The elastic reaction force, F(δ), is reported versus the AFM tip indentation, δ, for the outer surface of mature ZP (triangle), outer surface of fertilized ZP (square), and inner surface of fertilized ZP (circles). **b**: Histogram of the Young modulus for the inner (white) and outer (gray) surfaces of immature, mature and in vitro fertilized ZP, with standard deviation.

## Materials and Methods

Cow oocytes collection, in vitro maturation and fertilization, and ZP isolation were performed according to the procedure already reported [8]–[9]. Briefly, ovaries were obtained from a total of 11 cows and heifers at a local abattoir and were transported in saline solution at 37°C to the laboratory within 2 h from slaughter. Cumulus-egg complexes (COCs) were isolated from sliced ovaries, placed in Petri dishes and washed several times in PBS. Only the COCs with an intact, unexpanded cumulus oophorus and evenly granulated cytoplasm were chosen for the experiment. The selected COCs (n = 220) were washed three times in oocyte collection medium, tissue culture Medium 199 (Gibco, Invitrogen, Milano, Italy) supplemented with 10% (w/v) heat-inactivated fetal bovine serum (FBS) (Gibco, Invitrogen). Some oocytes (n = 145) were matured to metaphase II in Medium199, buffered with bicarbonate and supplemented with 10% (w/v) FBS, 0.1 UI/ml follicle-stimulating hormone (FSH) and 10 UI/ml luteinizing hormone (LH), at 39°C for 22–24 h at 5% CO2. Cumulus expansion was considered as a normal feature of oocyte maturation. Three different samples of commercial frozen semen was used for fertilization purposes of 98 matured oocytes. Motile sperm separation was carried out by using the Percoll gradient technique. Sperm concentration was determined with a hemocytometer. After 22–24 h of maturation, the COCs were washed three times in Hepes synthetic oviductal fluid medium (H-SOF, Sigma Aldrich, Milano, Italy) and placed in four-well culture dishes containing pre-equilibrated fertilization Tyrode’s Albumin-Lactate-Pyruvate medium (TALP-IVF, Sigma Aldrich) supplemented with heparin (1.2 g/ml). Spermatozoa were then added at a final concentration of 1×106 cells/ml in 300 µl medium per well containing a maximum of 20 COCs. In vitro fertilization was accomplished by coincubating oocytes and sperm cells for 20 h at 39°C at 5% CO2. The second polar body expulsion, observed by contrast microscopy, was considered to be sign of fertilization, the pronuclei visualization in living bovine zygotes being scant due to the dense yolk material [10]. COCs were denuded by vortexing for 3 min and washed three times in H-SOF medium. Thereafter, ZP of denuded oocytes was isolated by aspirating the cells in a narrow-bore pipette.

### Atomic Force Spectroscopy (AFS)

The physical properties of ZP were investigated by performing AFS measurements using a SPMagic SX Atomic Force Microscope (Elbatech, Italy), maintaining the samples on glass coverslips in an aqueous environment (Dulbecco’s phosphate buffered saline), at 37°C [11]. ZP membranes were turned upside up or upside down on coverslips to evaluate outer and inner layers separately. The microscope probe consisted in an ultrasharp silicon nitride cantilever of calibrated force constant [12] with a tip radius of less than 10 nm (CSC 16, MikroMash, USA). The mechanical measurements were carried out by lowering the AFM tip onto the ZP surface at a pre-established rate, typically 3 µm/s, with an indentation (δ) of 100 nm. After contact, the deflection of the cantilever, Δ, was recorded as a function of the piezoelectric translator position, Z. Within the limits of small deformation, the Hertz contact theory can be applied to describe the elastic contact behavior [9] and the value of the Young’s modulus, a measure of the stiffness, can be determined. The reaction force of the membrane (F) is related to the Young’s modulus by:
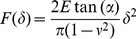
(1)where E is the Young’s modulus and F the reaction force of the membrane, calculated by applying the Hooke relation (F = kΔ) and indentation δ = Z−Δ. Several cantilevers with spring constants in the range between 0.05 and 0.12 N/m and a Poisson’s ratio of ν = 0.33 [13] were used. From electron microscopy image, the half-opening angle of tip apex, α, of 20° was determined. Mechanical responses for each experimental condition, i.e. both inner and outer layers of immature, mature and fertilized ZP, were determined and averaged over 20 different areas from each ZP.

Estimation of the energy necessary for a plastic deformation. In ZP, local mechanical stress can induce an elastic reversible reaction, or a plastic irreversible filaments deformation [8]–[9]. The energy of necessary to produce a plastic deformation can be calculated by:
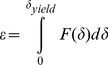
(2)where ε is the energy and δ_yield_ the critical indentation at which the ZP undergoes a plastic deformation.

**Figure 2:**
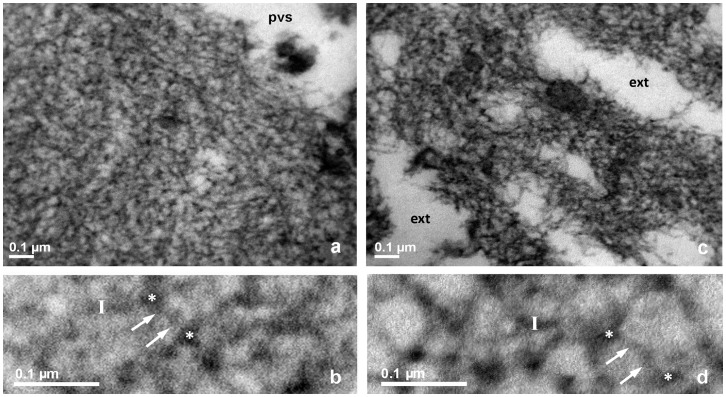
TEM of bovine ZP, immature oocyte, RR-saponin technique. **a**, **b**: inner layer; **c**, **d**: outer layer. In panels **a** and **c**, pvs: perivitelline space; ext: external ZP surface, 33.000 X. In panels **b** and **d**, closer views of **a** and **c**, respectively, arrows point to filaments whose length is measured between two knots, marked by white stars. Bars represent filament thickness, 119.000 X.

**Figure 3:**
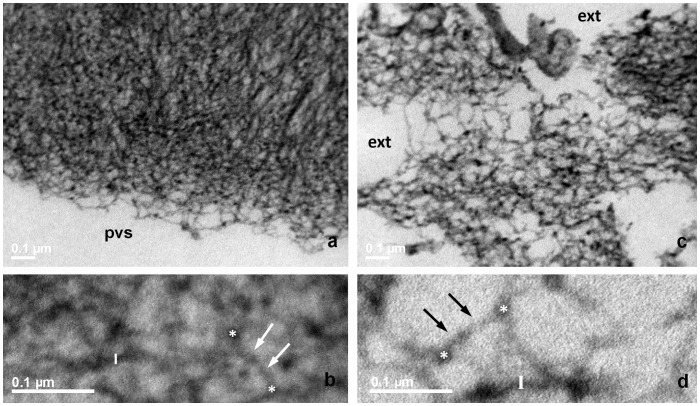
TEM of bovine ZP, mature oocyte, RR-saponin technique. **a**, **b**: inner layer; **c**, **d**: outer Layer. pvs: perivitelline space. In panels **a** and **c**, pvs: perivitelline space; ext: external ZP surface, 33.000 X. In panels **b** and **d**, closer views of **a** and **c**, respectively, arrows point to filaments whose length is measured between two knots, marked by white stars. Bars represent filament thickness, 119.000 X.

**Figure 4:**
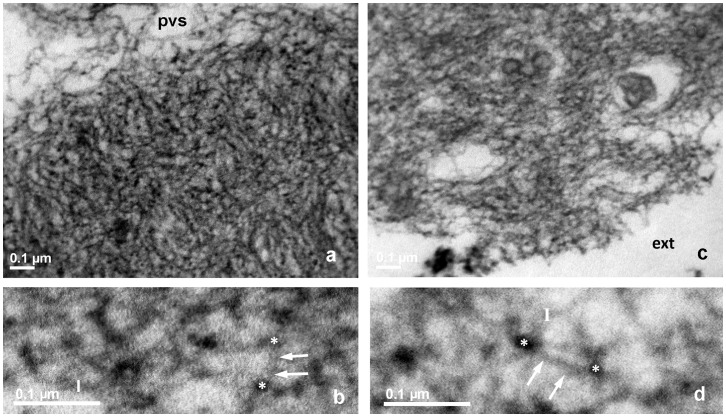
TEM of bovine ZP, fertilized oocyte, RR-saponin technique. **a**, **b**: inner layer; **c**, **d**: outer layer. pvs: perivitelline space. In panels **a** and **c**, pvs: perivitelline space; ext: external ZP surface, 33.000 X. In panels **b** and **d**, closer views of **a** and **c**, respectively, arrows point to filaments whose length is measured between two knots, marked by white stars. Bars represent filament thickness, 119.000 X.

### Transmission Electron Microscopy (TEM)

Bovine immature, in vitro matured and fertilized oocytes (n = 10 each) were fixed in 1% (v/v) glutaraldehyde in 0.1 M cacodylate buffer (pH 7.4) and stored at 4°C until processed. Samples were washed (four washes, 25 min each) using a 0.02% saponin solution and 1.0% ruthenium red (RR) in cacodylate buffer, then postfixed with a solution of 1.0% osmium tetroxide containing 0.02% saponin and 0.75% RR in cacodylate buffer for 2 hours [14], [15]. After postfixation, samples were dehydrated in ethanol and embedded in an epoxydic resin. Ultrathin sections (80–90 nm; silver-gold light interference) were obtained with a diamond knife using a Reichert ultramicrotome, and mounted in copper grids. Some ultrathin sections were stained with lead citrate [16], while others were examined without further staining. Observations were made with a Zeiss EM10 electron microscope operating at 80 kV.

For the image analysis, sections were made at the midpoint of the oocytes, in order to minimize bias due to tangential sectioning. Mean thickness of filaments (FT) was measured in the middle of the filament in the outer and inner portions of the ZP. Reported measures were averaged over 60 different areas, of 1.65 µm^2^ each, for each of the ten samples investigated [17]. Density of filaments network (large or tight meshed network) was evaluated by counting the length of filaments (FL) between two adjacent knobs both in the outer and inner portions of the ZP averaged over 60 different areas (1.65 µm^2^ each) for each of the ten samples investigated [18].

**Figure 5:**
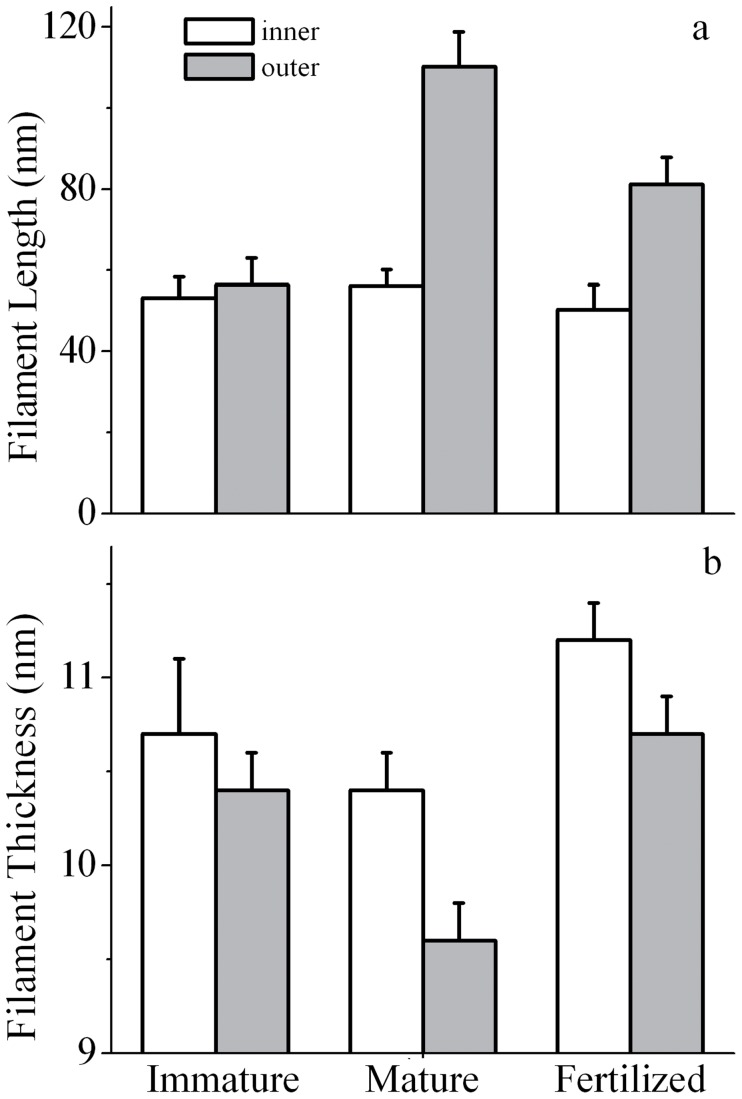
Ultrastructural characterization of ZP. Histogram of filament length (**a**) and thickness (**b**) computed from TEM images, with standard deviation, for the inner (white) and outer (gray) surfaces of immature, mature and in vitro fertilized ZP.

**Table 1:** Taxonomy distribution of genes in the training dataset.

Filament’s length (FL) and thickness (FT), number of measures (N), mean±SD					
Immature oocyte (ImO)		In vitro matured oocyte (MO)		Fertilized oocyte (FO)	
Inner ZP	Outer ZP	Inner ZP	Outer ZP	Inner ZP	Outer ZP
FL (N = 11)	FL (N = 11)	FL (N = 11)	FL (N = 11)	FL (N = 11)	FL (N = 11)
FT (N = 11)	FT (N = 11)	FT (N = 11)	FT (N = 11)	FT (N = 11)	FT (N = 11)
FL 53.1±3.2 nm	FL 57.1±4.2 nm	FL 56.5±2.1 nm	FL 111.8±5.1 nm	FL 50.1±2.9 nm	FL 81.2±6.0 nm
FT 10.7±0.1 nm	FT10.4±0.2 nm	FT 10.4±0.2 nm	FT 9.6±0.2 nm	FT 11.2±0.2 nm	FT 10.7±0.2 nm

Student’s t-test		
FL inner vs. outer T = 2.55 p = 0.01	FL inner vs. outer T = 33.25 p<0.0001	FL inner vs. outer T = 14.31 p< 0.0001
FT inner vs. outer T = 4.45 p = 0.0002	FT inner vs. outer T = 9.38 p <0.0001	FT inner vs. outer T = 5.86 p<0.0001

ANOVA test			
FL inner ZP	FT inner ZP	FL outer ZP	FT outer ZP
F = 14.67 P<0.0001	F = 59.89 P<0.0001	F = 284.38 P<0.0001	F = 88.91 P<0.0001

Post hoc Tamhane’s test			
FO vs. ImO P = 0.0321	FO vs. ImO P<0.0001	FO vs. ImO P<0.0001	FO vs. ImO P = 0.0022
FO vs. MO P<0.0001	FO vs. MO P<0.000	FO vs. MO P<0.0001	FO vs. MO P<0.0001
ImO vs. MO P = 0.008	ImO vs. MO P = 0.002	ImO vs. MO P<0.0001	ImO vs. MO P<0.0001

Measures were obtained on digital images, at original magnification 119.000 X, by using a Gatan digital micrograph apparatus (Gatan camera model 782 ES 500, equipped with Gatan microscopy suite 1.7.1 digital images acquisition software - Gatan Inc., Pleasanton, California, USA). Measurement of filaments length was made by the “Line ROI” function only considering planar filaments with a clearly visible origin and end between two adjacent connecting knobs. Filament thickness was calculated by using the same function, with reference to their middle portion; also in this case only filaments entirely visible in their whole length were considered for evaluation.

### Statistical Analysis

Data were reported as mean ± SD. The study variables are normally distributed. Differences between groups in comparison were analyzed by Student’s t test and Anova test. The presence of variance heterogeneity stressed by the Levene’s test and Welch’s test of equality of means, has led to the choice of the most conservative Tamhane’s test for post hoc multiple comparisons. Significance was defined as p<0.05. Statistical analysis was performed using MedCalc® 12.2.1 (Mariakerke, Belgium) and IBM-SPSS® statistics 20.0 (SPSS Italia Srl) softwares.

## Results

### ZP Biomechanics

In Fig. 1a we report three representative force-indentation curves, illustrating typical responses obtainable from different ZP samples, maintained in physiological conditions, i.e. in phosphate buffer and at 37°C. Reported plots originated from the outer layer of a mature (triangle) and fertilized (square) ZP, and from the inner layer of a fertilized ZP (circles). A relevant difference can be observed from mature and fertilized ZP, this last being also a little stiffer in the inner layer.

Complete data for all samples of the Young modulus, E, calculated from the force indentation curves following Eq. 1, are reported in Fig. 1b, with standard deviation. In immature oocytes, the E values of outer (E = 121±13 kPa) and inner (E = 118±16 kPa) layers were extremely similar (p = 0.6). Maturation of oocytes was paralleled by a significant decreased in E value, which affected both the outer (E = 20±3 kPa) (p<0.0001, T = 33.85 vs. immature) and inner (E = 32±9 kPa) (p<0.0001, T = 20.95 vs. immature) layers indicating a considerably softening of the whole ZP, more pronounced in the outer layer (p<0.001). In ZP from fertilized oocytes we observed a significant increase in E values, involving both the outer (E = 115±13 kPa) (p<0.0001, T = 31.84 vs. mature) and the inner layer (E = 140±14 kPa) (p<0.0001, T = 29.02 vs. mature) (Fig. 2C). Therefore, while the changes induced by fertilization were impressive, the reported difference between the two layers, although significant (p<0.001), was far less important.

### Energy Necessary to Displace ZP Filaments

The calculated energy necessary for a plastic ZP transition, i.e. for an irreversible filament deformation, resulted of (3.8±0.7) 10^−16^ J and (6.4±0.6) 10^−16^ J, in the outer layer of mature and fertilized samples, respectively.

### ZP Ultrastructure

TEM examination of the ZP from immature, mature and fertilized oocytes revealed a fine network of filaments anastomosed with each other and oriented in several different directions, sometimes forming small, rounded structures at the intersections. The filament arrangement was different between the inner and outer layers of the ZP and among the various maturation stages/fertilization of the oocytes studied.

#### The inner layer

The inner surface of immature oocytes (Figs. 2a, b) was homogeneous and regular, composed of a tightly meshed network of filaments, whose mean average length (FL) and thickness (FT) were of 53.1±3.2 nm and 10.7±0.1 nm, respectively. Slight but significant modifications in the filaments network organization and structure could be detected in mature (FL = 56.5±2.1 nm and FT = 10.4±0.2; Figs. 3a, b), and fertilized oocytes (FL = 50.1±2.9 nm and FT = 11.2±0.2 nm (Figs. 4a, b)).

#### The outer layer

The outer ZP layer of immature oocytes showed an indented and irregular aspect, due to the presence of typical fenestrations (Fig. 2c). Superficial indentations appeared as formed by a meshed network of filaments similar to that observed in the inner layer (FL = 57.1±4.2 nm and FT = 10.4±0.2 nm) (Fig. 2c, d). Mature oocytes showed the same indented and irregular aspect but with an overall looser organization of longer and thinner filaments (FL = 111.8±9.6 nm and FT = 9.6±0.2 nm) (Fig. 3c, d). Fertilized ZP showed shorter and thicker filaments (FL = 81.2±6.0 nm and FT = 10.7±0.2 nm) (Fig. 4c, d).

Complete FL and FT data with statistical evaluations are reported in Table 1, and a whole visual representation is reported as histogram, in Fig. 5.

## Discussion

The combined analysis by AFS and TEM showed that the variations in biomechanical properties recorded through the entire thickness of the ZP during the processes of maturation and fertilization are substantiated by ultrastructural changes.

ZP biomechanical properties varied significantly among the examined conditions, with a relevant decrease in stiffness paralleling the achievement of maturation. On the other hand, fertilization led to an increased rigidity that involved the whole thickness of the ZP: really, the reported differences between the inner and outer surfaces, despite significant, were minor. This evidence contradicts theoretical expectations that foresee more pronounced variations in the inner side of the ZP, due to the direct exposition to cortical granules exudates [19].

Therefore, as a first consideration, we conclude that the high incidence of bovine IVF-related polyspermy is not due to an incomplete propagation of post-fertilization stiffening through the ZP depth.

Recent findings suggest that modification in the overall organization of ZP structure, rather than sperm binding to a single ligand, is the major contributor of sperm binding regulation in the mouse [20]. In this regard, it is plausible to suggest that the significantly increased rigidity of ZP documented by the higher value of the Young modulus may concur, together with a modified structure, to decrease sperm binding capacity.

In this paper, the use of the ruthenium red-saponin method allowed a correlation between biomechanical and ultrastuctural properties of the thin ZP filaments morphology as it prevented filaments shrinkage due to mechanical stress induced by dehydration [14], [15], [21], [22]. The ultrastructural hallmarks of ZP maturation and fertilization were significant changes in the filament length and thickness. Indeed, the long and thin protein filaments present in mature ZP turned shorter and thicker after fertilization, resembling the same morphological features present in immature ZP. The increase in filaments thickness likely reflects structural rearrangements of fiber proteins, fitting the previously reported increased in β-sheet content and in disulfide bonds in fertilized ZP [17], [23].

For a rationale interpretation linking the observed ZP stiffening to changes in length and thickness of its constituent filaments we can refer to the classical theory of polymer [24]. Indeed, an increased stiffness is expected for: 1) a smaller network mesh size, brought about by a reduced filaments length; and/or 2) an increased number of inter- and/or intra-filaments cross-links, causing an increased filaments thickness. Our data perfectly fit both these predictions.

An increased stiffness does not necessarily involve greater difficulties in sperm transit through ZP. Indeed, calculation of the force necessary to displace filaments of fertilized ZP, i.e. to induce the so called elastic-plastic transition, gave quite similar data for the outer layer of mature and fertilized ZP, of (3.8±0.7) 10^−16^ J and (6.4±0.6) 10^−16^ J, respectively. This evidence clearly argue against the hypothesis that ZP stiffening effectively seals up the ZP, thus preventing the occurrence of polyspermy through the hindrance of sperm transit. Our consideration is further supported by the recently calculated energy generated by a single pulse of the bovine sperm flagellum, of 4.6×10^−16^ J [25], very close to the energy necessary to transit the cervical mucus [26] and to displace ZP filaments. Possibilities hold that an insufficient physical stiffening could be attributed to: 1) a lack of prefertilization hardening by oviductal factors, suggested of relevance in mammals including humans [27]; 2) the reported defective or incomplete granules release in *in vitro* as compared to *in vivo* fertilization in cows [7].

In conclusion, combining two high resolution complementary techniques TEM and AFM [28]–[30], we demonstrated that, soon after bovine IVF, there is a significant stiffening of the mature ZP that involves the entire ZP thickness, spreading from the inner to the outer surface and is substantiated by significant modifications of filaments length and thickness that change the overall 3D ZP texture. Such an increased stiffness after fertilization may still affect sperm binding. On the contrary, the occurrence of polyspermy well fits the absence of significant changes in the ZP elastic-plastic transition.

## References

[1] Hoodbhoy T, Dean J (2004) Insights into the molecular basis of sperm-egg recognition in mammals. Reproduction 127: 417–422. 15047932 10.1530/rep.1.00181

[2] Jovine L, Darie CC, Litscher ES, Wassarman PM (2005) Zona pellucida domain proteins. Annu Rev Biochem 74: 83–114. 15952882 10.1146/annurev.biochem.74.082803.133039

[3] Noguchi S, Yonezawa N, Katsumata T, Hashizume KI, Kuwayama M, et al (1994) Characterization of the zona pellucida glycoproteins from bovine ovarian and fertilized eggs. Biochim Biophys Acta 1201: 7–14. 7918585 10.1016/0304-4165(94)90143-0

[4] Sun QY (2003) Cellular and Molecular Mechanisms Leading to Cortical Reaction and Polyspermy Block in Mammalian Eggs. Microsc Res Tech 61: 342–348. 12811739 10.1002/jemt.10347

[5] Coy P, Cánovas S, Mondéjar I, Saavedra MD, Romar R, et al (2008) Oviduct-specific glycoprotein and heparin modulate sperm–zona pellucida interaction during fertilization and contribute to the control of polyspermy. Proc Natl Acad Sci USA 105: 15809–15814. 18838686 10.1073/pnas.0804422105 PMC2572915

[6] Coy P, Grullon L, Canovas S, Romar R, Matas C, et al (2008) Hardening of the zona pellucida of unfertilized eggs can reduce polyspermic fertilization in the pig and cow. Reproduction 135: 19–27. 18159080 10.1530/REP-07-0280

[7] Hyttel P, Callesen H, Greve T (1989) A comparative ultrastructural study of in vivo versus in vitro fertilization of bovine oocytes. Anat Embryol (Berl) 179: 435–442. 2729606 10.1007/BF00319585

[8] Papi M, Brunelli R, Sylla L, Parasassi T, Monaci M, et al (2010) Mechanical properties of zona pellucida hardening. Eur Biophy J 39: 987–992. 10.1007/s00249-009-0468-3 19471918

[9] Papi M, Sylla L, Parasassi T, Brunelli B, Monaci M, et al.. (2009) Evidence of elastic to plastic transition in the zona pellucida of oocytes using atomic force spectroscopy. App Phys Lett 94: 153902, 1–3.

[10] Minhas BS, Capehart JS, Bowen MJ, Womack JE, McCrady JD, et al (1984) Visualization of pronuclei in living bovine zygotes. Biol Reprod 30: 687–91. 6722241 10.1095/biolreprod30.3.687

[11] Papi M, Arcovito G, Vassalli M, Tiribilli B, De Spirito M (2012) Fluids viscosity determination by using uncalibrated atomic force microscopy cantilevers. Appl. Phys. Lett. 88: 194102.

[12] Papi M, Maulucci G, Arcovito G, Paoletti P, Vassalli M, et al.. (2008) Detection of microviscosity using uncalibrated atomic force microscopy cantilevers. Appl. Phys. Lett. 93, 124102.

[13] Boccaccio A, Frassanito MC, Lamberti L, Brunelli R, Maulucci G, et al.. (2012) Nanoscale characterization of the biomechanical hardening of bovine zona pellucida. J R Soc Interface. doi: 10.1098/rsif20120269; published ahead of print June 6, 1742–5662. 10.1098/rsif.2012.0269 PMC3479902 22675161

[14] Familiari G, Nottola SA, Macchiarelli G, Micara G, Aragona C, et al (1992) Human zona pellucida during in vitro fertilization: An ultrastructural study using saponin, ruthenium red, and osmium-thiocarbohydrazide. Mol Reprod Dev 32: 51–61. 1381198 10.1002/mrd.1080320109

[15] Familiari G, Relucenti M, Heyn R, Micara G, Correr S (2006) Three dimensional structure of the zona pellucida at ovulation. Microsc Res Tech 69: 415–426. 16703610 10.1002/jemt.20301

[16] Reynolds ES (1963) The use of lead citrate at high pH as an electron opaque stain in electron microscopy. J Cell Biol 17: 208–212. 13986422 10.1083/jcb.17.1.208 PMC2106263

[17] Kwamoto K, Ikeda K, Yonezawa N, Noguchi S, Kudo K, et al (1999) Disulfide formation in bovine zona pellucida glycoproteins during fertilization: evidence for the involvement of cystine cross-linkages in hardening of the zona pellucida. J Rep Fert 117: 395–402. 10.1530/jrf.0.1170395 10690208

[18] Garg SK, Valente E, Greco E, Santucci MB, De Spirito M, et al (2006) Lysophosphatidic acid enhances antimycobacterial activity both in vitro and ex vivo. Clin Immunol 121: 23–28. 16875878 10.1016/j.clim.2006.06.003

[19] Green DPL (1997) Three-dimensional structure of the zona pellucida. Rev Reprod 2: 147–156. 9414478 10.1530/ror.0.0020147

[20] Gahlay G, Gauthier L, Baibakov B, Epifano O, Dean J (2010) Gamete recognition in mice depends on the cleavage status of an egg’s zona pellucida protein. Science 329: 216–219. 20616279 10.1126/science.1188178 PMC3272265

[21] Familiari G, Heyn R, Relucenti M, Sathananthan H (2008) Structural changes of the zona pellucida during fertilization and embryo development. Front Biosci 13: 6730–6751. 18508691 10.2741/3185

[22] Familiari G, Heyn G, Relucenti M, Nottola SA, Sathananthan AH (2008) Ultrastructural dynamics of human reproduction, from ovulation to fertilization and early embryo development. Int Rev Cytol 249: 53–142. 10.1016/S0074-7696(06)49002-1 16697282

[23] Maulucci G, Papi M, Arcovito G, De Spirito M (2011) The thermal structural transition of α-crystallin inhibits the heat induced self-aggregation. PLoS One 6: e18906. 21573059 10.1371/journal.pone.0018906 PMC3090392

[24] De Gennes PG (1985) Scaling Concepts in Polymer. Ithaca: Cornell University Press.

[25] Allen MJ, Rudd RE, McElfresh MW, Balhorn R (2010) Time-dependent measure of a nanoscale force-pulse driven by the axonemal dynein motors in individual live sperm cells. Nanomedicine 6: 510–515. 20060073 10.1016/j.nano.2009.12.003

[26] Brunelli R, Papi M, Arcovito G, Bompiani A, Castagnola M, et al (2007) Globular structure of human ovulatory cervical mucus. FASEB J 21: 3872–3876. 17606809 10.1096/fj.07-8189com

[27] Avilés M, Gutiérrez-Adán A, Copy P (2010) Oviductal secretions: will they be key factors for the future ARTs? Mol Hum Reprod 16: 896–906. 20584881 10.1093/molehr/gaq056

[28] Tiribilli B, Bani D, Quercioli F, Ghirelli A, Vassalli M (2005) Atomic force microscopy of histological sections using a chemical etching method. Ultramicroscopy 102: 227–232. 15639354 10.1016/j.ultramic.2004.10.003

[29] Parasassi T, De Spirito M, Mei G, Brunelli R, Greco G, et al (2008) Low density lipoprotein misfolding and amyloidogenesis. FASEB J 22: 2350–2356. 18292214 10.1096/fj.07-097774

[30] De Spirito M, Brunelli R, Mei G, Bertani FR, Ciasca G, et al (2006) Low density lipoprotein aged in plasma forms clusters resembling subendothelial droplets: Aggregation via surface sites. Biophys J 90: 4239–4247. 16533854 10.1529/biophysj.105.075788 PMC1459520

